# ASG-YOLOv5: Improved YOLOv5 unmanned aerial vehicle remote sensing aerial images scenario for small object detection based on attention and spatial gating

**DOI:** 10.1371/journal.pone.0298698

**Published:** 2024-06-03

**Authors:** Houwang Shi, Wenzhong Yang, Danni Chen, Min Wang

**Affiliations:** School of Information Science and Engineering, Xinjiang University, Urumqi, China; VIT-AP Campus, INDIA

## Abstract

With the accelerated development of the technological power of society, aerial images of drones gradually penetrated various industries. Due to the variable speed of drones, the captured images are shadowed, blurred, and obscured. Second, drones fly at varying altitudes, leading to changing target scales and making it difficult to detect and identify small targets. In order to solve the above problems, an improved ASG-YOLOv5 model is proposed in this paper. Firstly, this research proposes a dynamic contextual attention module, which uses feature scores to dynamically assign feature weights and output feature information through channel dimensions to improve the model’s attention to small target feature information and increase the network’s ability to extract contextual information; secondly, this research designs a spatial gating filtering multi-directional weighted fusion module, which uses spatial filtering and weighted bidirectional fusion in the multi-scale fusion stage to improve the characterization of weak targets, reduce the interference of redundant information, and better adapt to the detection of weak targets in images under unmanned aerial vehicle remote sensing aerial photography; meanwhile, using Normalized Wasserstein Distance and CIoU regression loss function, the similarity metric value of the regression frame is obtained by modeling the Gaussian distribution of the regression frame, which increases the smoothing of the positional difference of the small targets and solves the problem that the positional deviation of the small targets is very sensitive, so that the model’s detection accuracy of the small targets is effectively improved. This paper trains and tests the model on the VisDrone2021 and AI-TOD datasets. This study used the NWPU-RESISC dataset for visual detection validation. The experimental results show that ASG-YOLOv5 has a better detection effect in unmanned aerial vehicle remote sensing aerial images, and the frames per second (FPS) reaches 86, which meets the requirement of real-time small target detection, and it can be better adapted to the detection of the weak and small targets in the aerial image dataset, and ASG-YOLOv5 outperforms many existing target detection methods, and its detection accuracy reaches 21.1% mAP value. The mAP values are improved by 2.9% and 1.4%, respectively, compared with the YOLOv5 model. The project is available at https://github.com/woaini-shw/asg-yolov5.git.

## 1 Introduction

**Fig 1:**
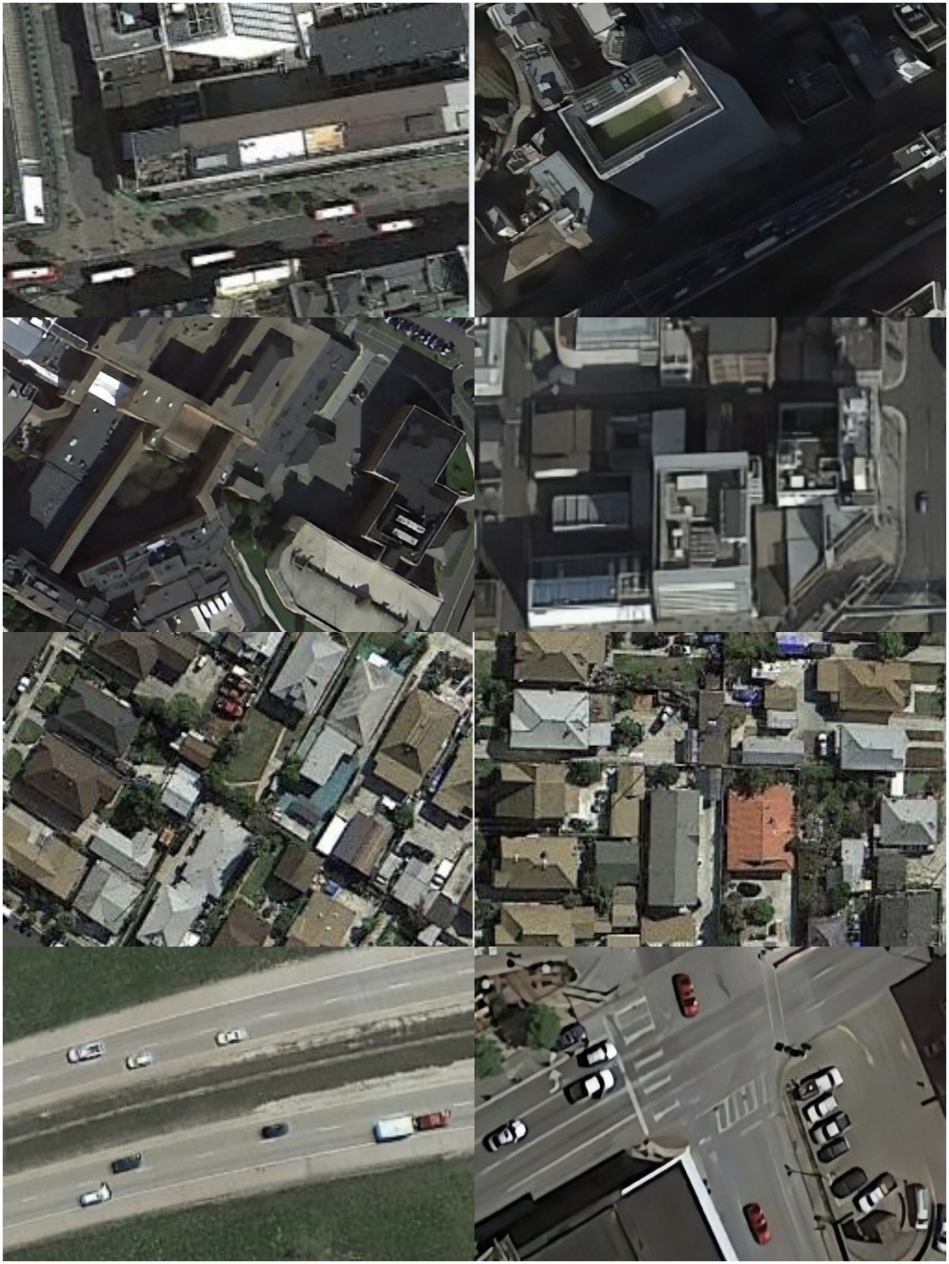
Images taken by the UAV show different problems of weak target detection. a) the problem of target occlusion in the images taken by the UAV; b) the problem of target recognition arising from the images taken by the UAV in different environments; c) the factors such as blurring and exposure of the images taken by the UAV at different speeds of motion affect target detection; d) the problems of different scales and many dense types of targets arising from images taken by UAVs flying at different altitudes. Fig 1 is attributed to the NWPU-RESISC database and are available from the NWPU-RESISC database (url(s) https://tensorflow.google.cn/datasets/catalog/resisc45).

With the rapid development of AI technology and deep learning, small target detection has progressed rapidly [1, 2], and models in the direction of small target detection have emerged. Small targets of different scales in images under aerial photography of unmanned aerial vehicles (UAV) have problems such as difficult recognition and multi-scale detection, category ambiguity, etc. In order to solve these problems, a small target detection model is introduced. UAV remote sensing aerial photography is widely used in road monitoring targets, photography, life images, agricultural development, and other kinds of applications; for example, UAVs remote sensing shoot pedestrians and vehicles on the road, and among the vehicles, there are large vehicles and small vehicles that need to be identified and detected in real-time. This research uses a small target detection model under deep learning to identify and analyze them through the real-time images captured by the UAV. The drone flight height produces pictures of target scales and the kinds of targets at different scales. There are no particularly weak targets in general images [3]. At the same time, there are different kinds of weak targets in aerial images, and the model completes the work of artificial intelligence through computational analysis. The detection of small targets in UAV remote sensing aerial images has the following three main problems: 1) UAV remote sensing aerial images need to be detected in real-time, and the images taken at high altitudes will have particularly weak targets that are easy to detect when there is a lack of targets to be detected and wrong detection targets; 2) there are a large number of different kinds of targets in the UAV remote sensing aerial photography images, which need to be divided into different kinds and detect different types of weak targets, some of which tend to overlap and be covered up by some unimportant or important kinds, which will cause particular problems for detection and identification; 3) the UAV remote sensing aerial photography images will appear at night and during the day, as well as various environmental factors such as lighting, making it difficult to identify and detect weak targets as well. The above three problems can be seen through the image comparison in Fig 1.

To address the above problems, this paper proposes the ASG-YOLOv5 model, designs the dynamic contextual attention module to solve the influence of dense scenes and blurred exposure scenes on small target detection and improve the model’s attention to small targets, and excessive space gating bidirectional filtering modules are designed to solve the impact of target occlusion and excessive redundant target interference on small target detection; The Normalized Wasserstein Distance [4] and CIoU [5] regression loss function are used to measure the similarity between the regression frames better and optimize the smoothing of the small target localization difference, which improves the accuracy and effectiveness of the model for small target detection. The model was extensively experimented on VisDrone2021 dataset [6] and AI-TOD dataset [7] and achieved better results than other models. In VisDrone2021-DET-val dataset and AI-TOD-test dataset, the mAP values reached 21.1% and 26.1%, respectively. A visual comparison was performed in the NWPU-RESISC dataset [8] and better detection results were obtained. The ASG-YOLOv5 model is more effective in detecting weak targets under UAV remote sensing aerial photography, and it can recognize more categories of weak targets and images of different scales.

The contribution of this paper is specified as follows.

- A dynamic contextual attention module (DCA) is proposed to filter redundant information using information bottlenecks, dynamically assign weights by target feature scores to enhance the extraction of global information, and output feature context information through channel dimensions to enable the network to focus better and extract useful target features.
- A spatially gated filtering multi-directional weighted fusion module (SGM) is proposed to enhance the characterization of small target categories by suppressing large target categories through spatially gated units, introducing global attentional up-sampling to increase the small target feature information in the underlying feature layer, and performing a weighted fusion of feature layers at different scales to enhance the characterization of small target categories.
- This research optimizes the regression loss function of the model by introducing Normalized Wasserstein Distance loss, which is combined with the CIoU loss function to increase the smoothness of the positional difference of the target frames, increase the similarity between the small target categories, and ultimately improve the model’s detection accuracy and effectiveness for small targets in UAV scenarios.
- On the unmanned aerial vehicles remote sensing datasets VisDrone2021-DET-val and AI-TOD-test, the ASG-YOLOv5 model designed in this paper achieves 21.1% mAP and 26.1% mAP values, respectively, which significantly improves the small target detection capability by 2.9% and 1.4% mAP values, respectively, compared with the baseline YOLOv5 model. A visual comparison was performed in the NWPU-RESISC dataset and better detection results were obtained. At the same time, the frames per second (FPS) reach 86, which meets the requirement of real-time small target detection in UAV remote sensing scenarios and has better accuracy and speed advantages than most UAV remote sensing target detection models.

The remaining chapters of this thesis are organized as follows: Section 2 describes the related work used to design the model used in this paper and its details. Section 3 presents the overall structure of the ASG-YOLOv5 model, the main modules designed, and the loss function. Section 4 presents the details of the experiments done by the model on the two UAV datasets, the comparison experiments, and the ablation experiments. Finally, Section 5 summarizes the results of the methodology study presented in this paper, methodological limitations, and the outlook of the next research.

## 2 Related work

As small target detection methods have evolved, their models’ accuracy in detecting small targets has gradually improved. However, the UAV remote sensing shooting scene has many target objects, and detection is still difficult. Hence, this paper introduces a redesigned model structure for small target detection that incorporates multi-scale feature fusion and data enhancement techniques for input images. These methods have improved the accuracy and effectiveness of small target detection in UAV scenes.

**Small Target Detection**. The initial violent approach to target detection is a sliding window in different directions, using classification to identify targets. Windows of different sizes and aspect ratios detect different target types at different observation distances. The existing models for small target detection can be divided into one-stage and two-stage models, and in 2014, Ross Girshick proposed R-CNN [9], the initial two-stage target detection model. R-CNN is a model for object detection based on a deep neural network, which has attracted the world’s attention for its excellent performance at that time. The R-CNN is a model for object detection based on deep neural networks, and it has been noted for its excellent performance at that time. It still uses selective search to select 2000 suggestion boxes; due to the different sizes of suggestion boxes, the obtained feature boxes need to be transformed to the same size by the ROI Pooling layer. Fast R-CNN [10] classifies and predicts the position size of boxes output by the convolutional neural network. The network finally uses SVD instead of a fully connected layer to improve the computational speed. In 2015, Kaiming He, Ross Girshick, et al. proposed the famous Faster R-CNN [11] algorithm, a breakthrough in proposing RPN (Region Proposal Network) network, using an anchor mechanism to link region generation with the convolutional network, abandoning the selective search. The algorithm has improved the detection speed to 17FPS (Frames Per Second) and achieved 70.4% detection results on the VOC2012 test set.

The one-stage models started with Wei Liu’s presentation of SSD at ECCV 2016 [12] and YOLO [13] presented by Joseph Redmon at the University of Washington in 2016. YOLO has significantly improved small target detection from the original YOLOv1 to YOLOv5 [14]. The main contribution of YOLO is the development of a real-time high-performance target detection framework for one-stage detection, which predicts the location and class of targets in an image by simply feeding the image into the network at once. In contrast to YOLOV4 [15], proposed by Glenn Jocher in 2020, YOLOv5 enhances each batch of training data by passing it through a data loader. The data loader performs three types of data enhancement: scaling, color space adjustment, and mosaic enhancement. YOLOv5 and YOLOV4 use CSPDarknet as the backbone to extract rich target feature information from the input image. CSPNet solves the problem of duplicating gradient information for network optimization in the backbone of other large convolutional neural network frameworks. This is done by integrating the gradient changes into the feature map from beginning to end, reducing the number of parameters and FLOPS values of the model, ensuring both inference speed and accuracy, and reducing the model size [15]. For the aerial pictures taken by UAVs, the small target category is more difficult to identify. Hence, this paper chooses to use the faster YOLOv5 model as the base model, adding an attention mechanism and gating unit to filter and detect small targets in the UAV view, which can detect weak targets in more real-time and more accurately. Eventually, there was a significant improvement in accuracy and mAP for the improved YOLOv5 model in several experiments.

**Feature fusion and enhancement at different scales**. Multi-scale takes sample information of signals with different granularity and effectively improves the detection of weak targets by fusion and enhancement of multi-scale features. In the field of target detection, the SSD (Single Shot MultiBox Detector) [12] network proposed by Wei Liu in 2016 is the initial network for target detection direction using different scale feature fusion. Later, the FPN proposed in 2017 [16] led to a significant improvement in the fusion of scale features in the small target detection direction, and there have been researchers who have continuously improved the FPN since then. The PAFPN [17] structure used in the subsequent YOLOv5 [14] better transfers the target information from different feature layers, from top to bottom and from bottom to top. The subsequent BiFPN [18] structure saves the connection transfer between some of these feature layers and introduces weights to learn the importance between the different feature layers of the input. The subsequent AF-FPN [19] structure, proposed in 2021, uses the adaptive attention module (AAM) to reduce the absence of contextual information in the high-level feature maps and the feature enhancement module (FEM) to enhance the representational power of the pyramid structure. The network mentioned above structures are relatively effective in fusing and enhancing features at different scales but need more simultaneous fusion and enhancement of feature information, and the communication between feature layers is complicated, which can easily disrupt the model’s focus on small target detection.

In this paper, this research improves FPN by adding a new spatial gated filtering multi-directional weighted fusion module to provide a better-weighted fusion of features at different scales. At the same time, by filtering out the redundant information of other feature layers and increasing the small-target pixel information of the underlying features, the model pays more attention to the information of the weak and small targets to enhance the characterization ability of the small-target features.

**Data Enhancement**. The aerial images taken by drones as the dataset used for experiments will have the problems of a small number of small targets in the images and a small percentage of tiny target labeled areas, resulting in uneven distribution of small targets in the images, and therefore poor generalization ability in terms of location. In the actual test images, however, small targets are likely to appear in areas that did not appear in the training set [20] and are difficult to detect. The images contain a large number of background categories. At the same time, data enhancement enables the dataset to be extended to increase the effect of the model in detecting small targets, which essentially solves some of the problems contained in the original dataset and improves the robustness of the model to different data. So, this research chose to perform data augmentation on the dataset. Now, there are many processing methods; researchers use Copy-Pasting [21] (Copy-Pasting strategy), meaning that the small target is pasted to any location in the image. A new annotation is generated, and the pasted small target can be randomly transformed (scaled, folded, rotated, etc.). This process ensures that the Context in which the small target is located is appropriate [20]. In this way, by increasing the number of small targets in each image, the number of matched anchors increases, which improves the contribution of small targets to the loss calculation in the training phase. Some researchers use Cutouts [22] to randomly select a square region of fixed size and then use all-0 padding. Meanwhile, Mosaic [15] improved on CutMix [23] by using four images for splicing while randomly selecting a region as the region of interest and splicing it, and passing the newly synthesized image into the neural network for learning as a way to improve the robustness and generalization of the model.

In this paper, this research uses Mosaic, Cutout, Copy-Past, and traditional data enhancement methods to simultaneously perform data enhancement on the dataset. Target detection dataset once the image data is enhanced (target box is changed), the labels need to be modified accordingly. These three methods are chosen because they can be easily embedded in the training framework of this research, help increase the model’s detection effect in scenarios with small targets and lack of target background, and are an excellent way to solve the unbalanced data set with small numbers and a proportion of samples.

## 3 Method

In this section, this research provided a thorough description of the compositional architecture of the ASG-YOLOv5 method designed in this paper. It elaborated on the details of the custom-designed Dynamic Contextual Attention Module (DCA), Spatial Gating Filtering Multi-directional Weighted Fusion Module (SGM), and NWD-CIoU Loss regression loss function. This research effectively improved the overall model performance for detecting small targets in UAV scenarios by leveraging the modules designed in this paper and network structure.

### 3.1 ASG-YOLOv5 model

**Fig 2:**
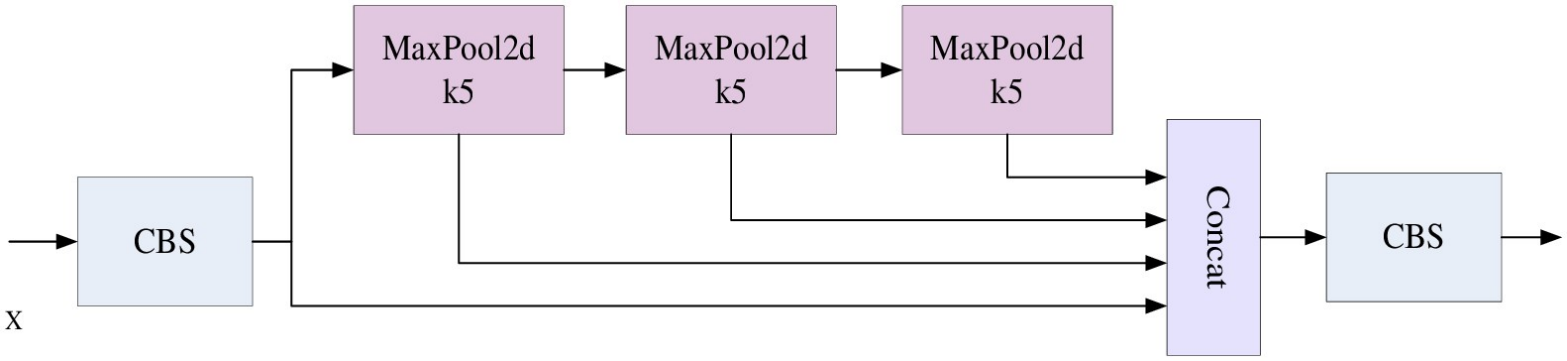
Detailed module diagram of Spatial Pyramid Pooling—Fast (SPPF). The CBS module denotes the convolution, the BN layer, and the SiLU activation function.

**Fig 3:**
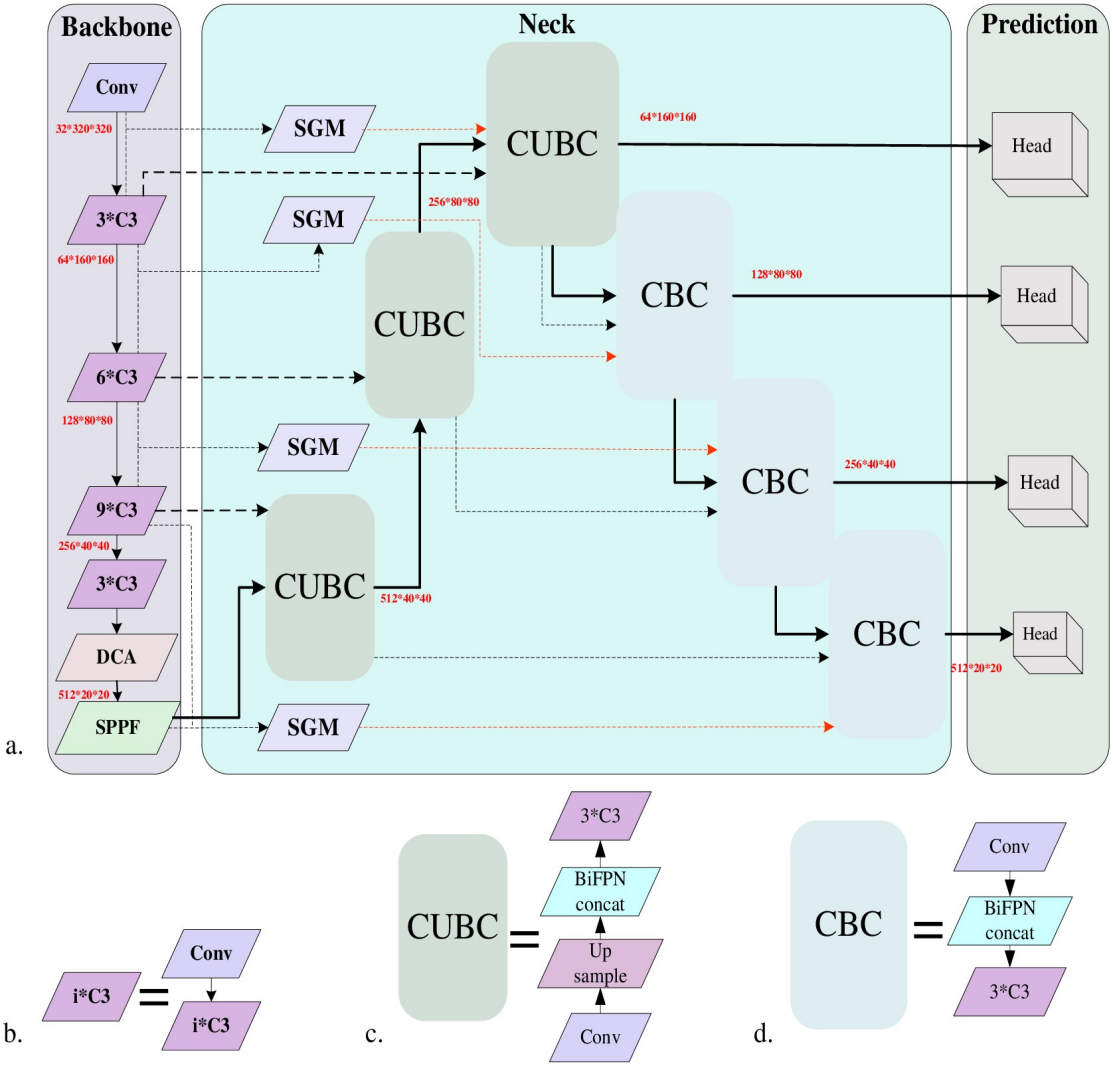
(a) ASG-YOLOv5 model structure. (b) C3 module. (c) Consists of a C3 layer, BiFPN fusion, upsampling operation, and a convolutional layer. (d) Consists of a convolutional layer, BiFPN fusion, and a C3 layer. Adding DCA module at the end with CSPDarknet53 of base model YOLOv5 as the backbone; introducing a new spatially gated filtered multi-directionally weighted fusion module, SGM, with PAFPN similar to base model YOLOv5 as the neck module.

In this paper, based on the design of Dynamic Contextual Attention Module (DCA) and Spatial Gating Filtering Multi-directional Weighted Fusion Module (SGM), this research incorporates the Dynamic Contextual Attention Module at the end of the backbone network, and at the end of the Spatial Gating Filtering Multi-directional Weighted Fusion Module (SGM) module is introduced in PAFPN [17]. First, the input feature maps are extracted from different scales by the DCA module and SPPF module at the backbone and end of the network to improve the network’s focus on useful target features and output the P2, P3, P4, and P5 feature layers. The detailed structure of spatial pyramid pooling—fast (SPPF) module is shown in Fig 2. The feature layers are up-sampled to obtain C5, C4, C3, and C2 feature layers. The Pi(i = 2,3,4) and Pi+1 feature layers extracted from the backbone network are filtered and localized to the small target category through the fusion of SGM module using spatial gating unit (SG) and BiFPN-Concat [18] in order to filter out the redundant feature-target information. Finally, the filtered feature layers are fused with Ci(i = 2,3,4) to obtain the final output feature layer Fi(i = 2,3,4). Finally, the output four-layer feature layer is used as the head for predicting weak and small targets. The overall structure and details of the relevant modules of the ASG-YOLOv5 model proposed in this paper are shown in Fig 3.

### 3.2 Dynamic Contextual Attention module (DCA)

**Fig 4:**
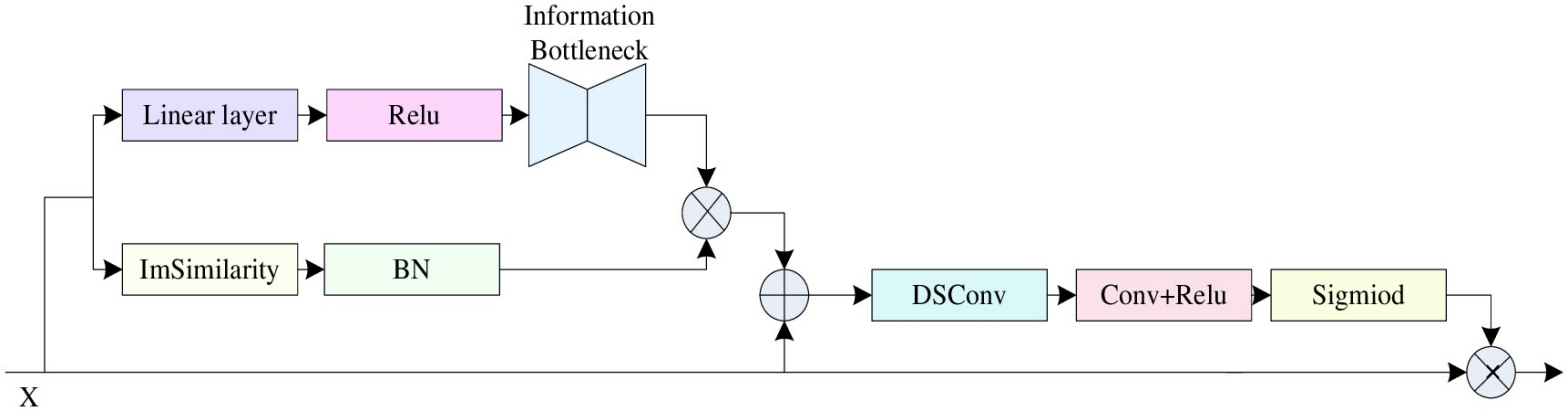
Detailed diagram of dynamic contextual attention module (DCA). Input feature x goes through a global contextual information extraction structure containing feature similarity score and information bottleneck structure to get global information; the global information is fused with the original input feature information, and the weights of different channels are recalibrated to adjust the channel dependency, and the obtained feature weights of different channels are multiplicatively weighted and fused with the input features.

Attention mechanisms are widely used in the field of vision, such as Vision Transformer (VIT) [24] and Swin-Transformer [25, 26], whose methods aim to make the network pay better attention to useful feature target information. There is very little small target information in the UAV remote sensing shooting dataset. The network has a low ability to pay attention, and the existing attention assigns weights from the channel and spatial dimensions to improve the overall attentional effect of the model. The existing attention mechanism cannot fully utilize the connection to global information in the feature layer, and the computational volume and parameters are huge, making the overall inference speed of the model slow. Based on the above problems, this research proposes a new dynamic contextual attention module—DCA, whose structure is shown in Fig 4, which firstly utilizes feature similarity scores to assign feature information weights dynamically, integrates global contextual feature information, in which this research adds an information bottleneck structure, reduces redundant information in the output features, and retains the most relevant global information of the input and output features, which reduces the impact of the introduced redundant information. The module reduces the introduction of additional parameters and network inference speed. Moreover, this research uses the separable depth convolution to fuse the local feature information to get the fused contextual feature information. As shown by Eq 1.

$$g=\left(\tau +\varphi \frac{S-\alpha }{\beta }\right)\left(Conv\left(\sum_{i=1}^{n}\frac{x_{i}}{n}\right)\right),$$ (1)

where x denotes the input feature, τ and φ denote the learnable scalar, respectively, S denotes the target feature information score, and α and β denote the mean and standard deviation of the target feature information score, respectively.

**Fig 5:**
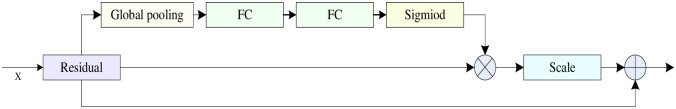
Detailed schematic of the Squeeze-and-Excitation module (SE). The input feature x is residual-connected, and a part of it is dimensionally compressed using global average pooling. It goes through two fully connected layers to predict each channel and get the importance of different channels. Then, a normalization operation is performed using the sigmoid activation function, and then feature fusion is performed with the other part of the input features.

Inspired by the Squeeze-and-Excitation Networks (SENet) [27, 28] network model shown in Fig 5, this research improves the generalization ability of the module by using channel attention to compute the weights of the fused contextual feature information on the channel dimension. The channel attention extracts the fused global information and input feature information using depth separable convolution (DSConv), which has a relatively low number of parameters and computational cost, and after the normalization layer and ReLU activation function, performs the weight normalization operation on the extracted fused features and multiply fuses the normalized weights with the features of different location information, as shown in Eq 2.


$$v_{i}=Sigmoid\left(Conv\left(ReLU\left(LN\left(Conv\left(DSConv\left(g+x\right)\right)\right)\right)\right)\right)*x,$$ (2)

This research adds the DCA module to the end of the backbone network of YOLOv5 [14] to ensure the inference time is manageable and the number of model parameters is manageable. In the feature extraction stage, the DCA module makes the backbone network more effective in extracting the feature information of the target by fully extracting the fusion context information and performing inter-channel computation on the fused information, which improves the ability of the network to focus on useful target features.

### 3.3 Spatial Gating Filtering Multi-directional Weighted Fusion Module (SGM)

**Fig 6:**
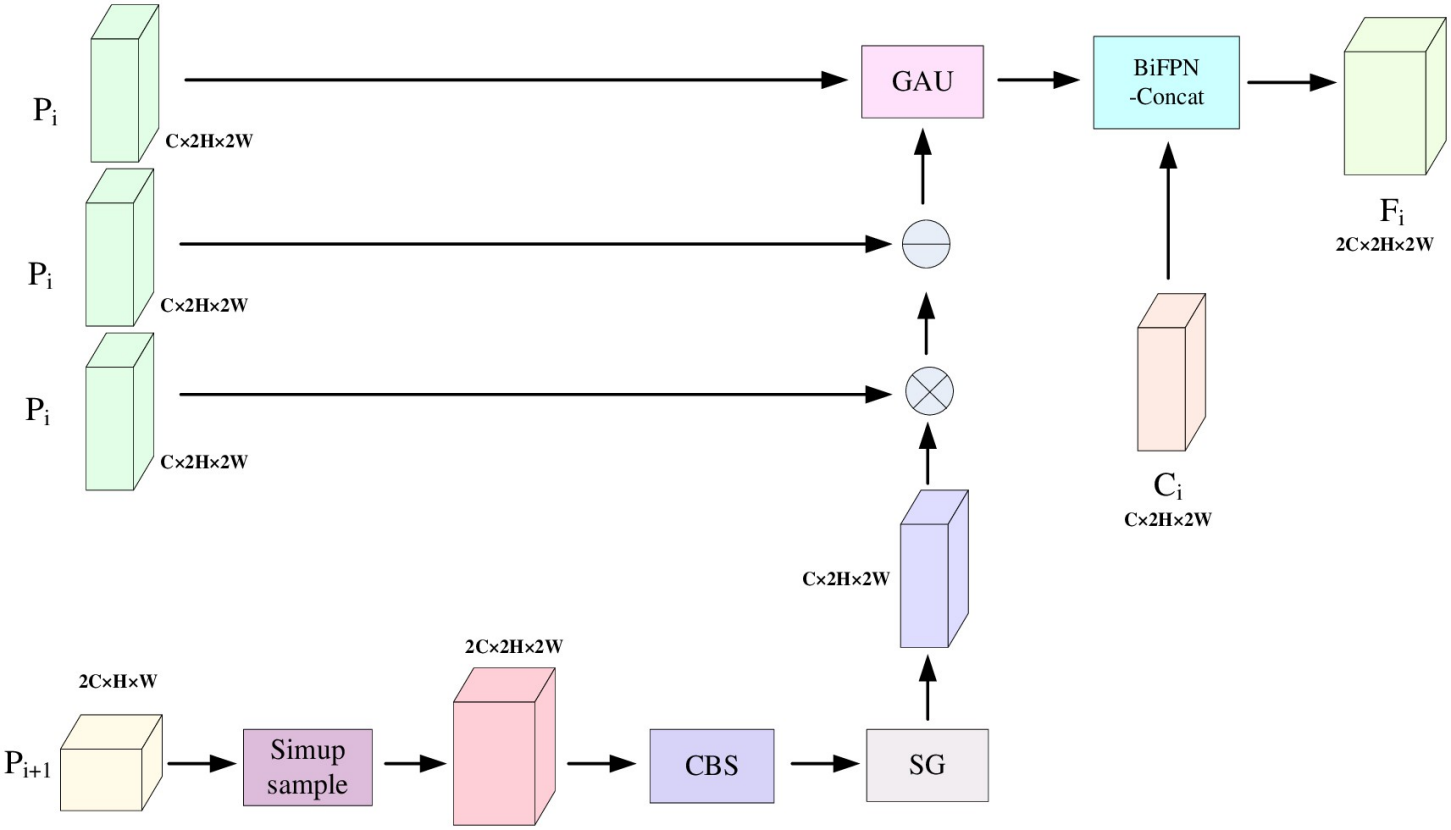
Spatial Gating Filtering Multi-directional Weighted Fusion Module (SGM) module structure. Simupsample is Content Aware Reassembly of Features (CARAFE), the CBS module consists of Conv layer, BN layer, Silu layer, SG is a spatial gating unit, GAU denotes Global Attention UpSample module, and the BiFPN-Concat module is the weighted feature fusion module.

In the UAV remote sensing aerial images, weak and small targets are mixed with large targets, which are difficult to recognize. The input image of the backbone network will get different feature layers, and different feature layers pay different attention to the details of small targets; the P5 feature layer, as a deep layer, exists more target semantic information and pays more attention to the details of the features of the target to be detected, and the P2 feature layer, as a shallow layer, has particularly low pixels of target information and pays more attention to the overall features of the target to be detected. At the same time, each feature layer contains different size targets, and the fusion of feature layers of different scales will produce redundant feature target information. In order to solve the above problems, this research designs a spatial gated filtering multi-way weighted fusion module (SGM), which filters out the interference of large targets and redundant information through spatial gated filtering units, enhances the small target pixel information of the underlying feature layer by global attentional up-sampling, and fuses the two neighboring feature layers in a weighted manner, so that the model can better extract the useful target feature information and focus on detecting the small target features. The main structural details are shown in Fig 6.

**Fig 7:**
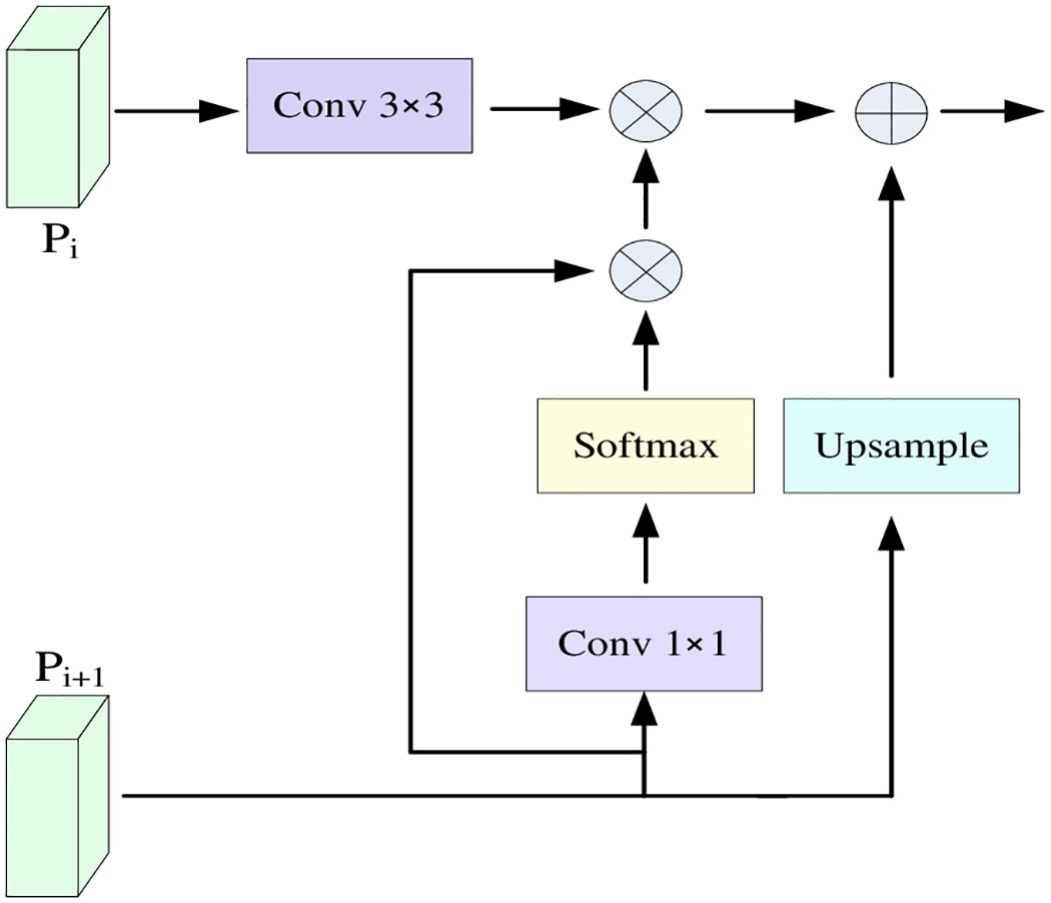
Detailed schematic of the Global Attention Upsample (GAU) module. Pi denotes the shallow feature layer, and Pi+1 denotes the deep feature layer.

The input Pi+1 (i = 2, 3, 4) feature layer is first subjected to a lightweight, universal upsampling operation: Content Aware Reassembly of Features (CARAFE) [29]. The upsampling kernel prediction module and feature recombination module are used respectively, introducing only a small amount of parameters and computing costs while obtaining a larger Receptive field to aggregate the context information of features. The feature information of this layer is then transformed and extracted through the CBS module, which consists of a Conv layer, a BN (batch normalization) layer, and an activation function Silu layer. Next, after the spatial gating unit SG, before multiplying the input features pixel by pixel, the ordinary convolution is replaced by using the dilated convolution [30, 31], which captures a larger range of contextual information by adjusting the dilation rate while keeping the convolution kernel size unchanged. This enables the dilated convolution to capture a broader range of semantic associations and better understand the global structure and contextual information in the image. Also by adjusting the dilation rate, a larger sensory field can be obtained without introducing additional parameters, thus reducing the number of parameters and computational complexity of the model to some extent. The use of multiple dilated convolutional layers to enrich the receptive field, as well as the final normalization operation using the ReLU activation function, can dynamically adjust the weights of the target features in the feature map in order to better capture the spatial information of the target object. Then, a point-by-point multiplication operation is performed with the Pi (i = 2,3,4) feature layer to increase the representation of small target features. The function obtained from the previous module is then subtracted point-by-point with the Pi(i = 2,3,4) feature layer to eliminate large target features and filter small target features. The output feature layer is then fused with the shallow feature layer using the Global Attention Upsample (GAU) [32] module to increase the small target pixel information of the underlying feature layer. The detailed structure diagram is shown in Fig 7. There is more small target semantic information in the deep feature layer; this research extracted the global context feature information from the new feature layer obtained after point-by-point subtraction by using 1×1 convolutional layer and Softmax function normalization operation and performing 3×3 convolutional processing with channel dimension on the shallow Pi (i = 2,3,4) feature layer. The global context information features of the deep layer are weighted and fused with the shallow features in the channel dimension. Finally, the deep feature layer is up-sampled and fused with the weighted shallow features to output the new feature layer information. The small target semantic information of the shallow feature layer is fused into the shallow feature layer to enhance the small target feature information so that the model can locate the small target information and valuable information more accurately when extracting features. Finally, the new feature layer is output with Ci(i = 2,3,4) feature layer by BiFPN-Concat [18]; it contains the connection of multi-directional different scale feature layers and the fusion of fast normalization. BiFPN-Concat adds weight coefficients on top of the concat and normalizes the weights to fuse different numbers of branching feature layers separately to fuse better feature layers of different scales and better focus on small targets.

### 3.4 Normalized Wasserstein Distance- Complete IoU Loss (NWD-CIoU)

Since small targets contain only a few pixel sizes, and at the same time, small target features lack appearance information, it is difficult for state-of-the-art target detection models to localize and identify small targets accurately. In the dataset captured in the UAV scenario, the small target category accounts for most of the data, making the existing model less accurate and less effective for target category detection. The existing IoU metrics in YOLOv5 are extremely sensitive to the positional difference of the small targets, which also leads to the poor accuracy of the small target detection. Therefore, in order to improve the model’s detection accuracy and effect for small targets, this research introduced Normalized Wasserstein Distance (NWD) [4], which is used as an evaluation method for small target detection through Wasserstein distance, and combined with CIoU [5] to optimize the existing loss function. Firstly, this research calculated the center distance, overlap rate, and aspect ratio between the target and the anchor frame in order to make the anchor frame regression loss more stable. The specific formula is expressed as follows.

$$CIoU=IoU-\frac{\rho^{2}\left(b,b^{gt}\right)}{c^{2}}-\alpha \nu ,$$ (3)

$$\alpha =\frac{\nu }{1-IoU+\nu },$$ (4)

$$\nu =\frac{4}{\pi^{2}}{\left(arctan\frac{w^{gt}}{h^{gt}}-arctan\frac{w}{h}\right)}^{2},$$ (5)

where ρ^2^(b, b^gt^) denotes the Euclidean distance between the centroids of the predicted and real frames, respectively, c denotes the diagonal distance of the minimum closure region, α denotes the equilibrium coefficient, ν denotes the aspect ratio of the anchor frame, w and h denotes the length and width of the anchor frame.

Meanwhile, this research modeled the 2D Gaussian distribution of the BBox box to more fully show the weights of different positional feature information, calculate the distance metric for the positional information of the two target boxes, and finally normalize to get the similarity metric value between 0–1. The formulas are expressed as shown in Eqs 6 and 7.

$$W_{2}^{2}\left(N_{a},N_{b}\right)=\|({\left[cx_{a},cy_{a},\frac{w_{a}}{2},\frac{h_{a}}{2}\right]}^{T},{\left[cx_{b},cy_{b},\frac{w_{b}}{2},\frac{h_{b}}{2}\right]}^{T}){\|}_{2}^{2},$$ (6)

Where $\text{A}=\left[cx_{a},cy_{a},\frac{w_{a}}{2},\frac{h_{a}}{2}\right]$ and $\text{B}=\left[cx_{b},cy_{b},\frac{w_{b}}{2},\frac{h_{b}}{2}\right]$ the Gaussian distribution modeling the two target frames is denoted as (*N*_*a*_, *N*_*b*_), $W_{2}^{2}\left(N_{a},N_{b}\right)$ denotes the difference measure between the target frames.

$$NWD\left(N_{a},N_{b}\right)=exp\left(-\frac{\sqrt{W_{2}^{2}\left(N_{a},N_{b}\right)}}{C}\right),$$ (7)

Where C denotes the value of the constant required to be associated with the data in the dataset.

This research did equal weight coefficients of the obtained metrics with the CIoU regression loss and added them as the final regression loss function value. As a result, this research proposed the NWD-CIoU Loss, whose formulaic expression is shown in Eq 8.

$$L_{NWD-CIoU}=\alpha *\left(1-CIoU\right)+\beta *\left(1-NWD\left(N_{a},N_{b}\right)\right),$$ (8)

where α, β denote the equilibrium coefficients of the regulation loss function.

This research optimized the loss function by Normalized Wasserstein Distance, which ensures the smoothness of the model for the positional differences and, at the same time, increases the similarity between the two target frames. Finally, the detection accuracy and effect of the model for small target features are effectively improved.

## 4 Results and discussion

This section demonstrated the effectiveness of the ASG-YOLOv5 method proposed in this paper by performing a comprehensive series of comparisons on the VisDrone2021 and AI-TOD datasets, and ablation experiments on the VisDrone2021-DET-val. First, this research described the VisDrone2021 and AI-TOD datasets used for the experiments, the parameters used for model training and testing, and the evaluation metrics used for validation. Subsequently, this research compared the validation results and visualization plots of the current state-of-the-art target detection methods on the VisDrone2021-DET-val and AI-TOD-test datasets. Finally, this research performed ablation studies for each of the proposed modules.

### 4.1 Experimental details

In this paper, this research used NVIDIA A40 GPUs (48G of video memory) for training and testing; the model code is written under Python 3.6 to implement the ASG-YOLOv5 model for target detection in UAV remote sensing scenarios based on the Pytorch 1.10 deep learning framework. The ASG-YOLOv5 model designed in this paper has many of the same parts as YOLOv5s, such as the backbone and neck, so the model also uses the weights of YOLOv5s. This research used 640×640 pixels for the input image size, set the batch size to 64, and set the epochs to 300. The training loss of the optimizer decreases faster than the SGD [33]. The initial learning rate lr0 is set to 0.005, and the periodic learning rate lrf is set to 0.1.

### 4.2 Experimental dataset

In this experiment, this research used the training and validation sets from the VisDrone2021 dataset [6] for training and testing, which contains ten categories, 6,471 images for the training set, and 1,610 images captured by different UAVs for the validation set, which cover a variety of aspects, such as different cities and their environments, people, and scene densities, etc. This research also used the AI-TOD dataset [7] for the training and test sets, which contain eight categories, 11214 aerial images from the training set and 14018 aerial images from the test set covering weak target objects such as cars, airplanes, pedestrians, etc. The pixels of the image are 12.8 pixels, much smaller than those of other aviation scene images. Also, this study uses the NWPU-RESISC dataset [8] for target detection visualization comparison. After the experiments, this research can see that the model designed in this paper better detects weak targets in UAV remote sensing aerial images and can better focus on and identify weak targets. During the experiment, this research used data enhancement to expand the data set to improve the robustness of the network. First, this research used traditional data enhancement to scale and rotate the image to crop and change the color gamut for adjustment. This research then used Cutout [22] randomly selected regions for cropping, Mosaic [15] method to perform a four-image puzzle, and the Copy-Paste [21] method to paste and copy the images to fuse them. After data enhancement, this research finally got good test results.

### 4.3 Evaluation metrics

This research chose mAP (average of all 10 IoU thresholds, ranging from [0.5: 0.95] for all categories of AP calculations), AP_50_, and AP_75_ as the judging results. mAP (AP_val_) was used to measure the overall accuracy rate.

AP_50_ calculates the average accuracy of all images in each category when IoU is set to 0.5 (accuracy at different recall points is averaged) and then averages over all categories. The expression can be written as

$$\text{AP}=\int_{0}^{1}\text{p}\left(\text{r}\right)\text{dr},$$ (9)

where r denotes recall and p denotes accuracy. mAP is the result of taking the average accuracy value between 0.5 and 0.95 for IoU and then taking the average value, and the expression can be written as

$$\text{mAP}=\frac{\sum_{\text{i}=1}^{\text{K}}{\text{AP}}_{\text{i}}}{\text{K}},$$ (10)

where K denotes the category.

Moreover, this research uses AP_vt_, AP_t_, AP_S_, AP_L_, and AP_M_ as the detection accuracy values of the model for minute, tiny, small, medium, and large targets; AP_S_ denotes average precision (AP) measurements for target frames with pixel areas less than 32^2^; AP_M_ denotes AP measurements for target frames with pixel areas between 32^2^ and 96^2^; AP_L_ denotes AP measurements for target frames with pixel areas greater than 96^2^. Since the AI-TOD dataset has minimal, tiny, and small targets, this research includes AP_vt_ and AP_t_ as the detection accuracy values for minute and tiny targets in evaluating the model using the AI-TOD dataset. AP_vt_ denotes the target detection accuracy value for pixels between 2 and 8, and AP_t_ denotes the target detection accuracy value for pixels between 8 and 16. The size and detection speed of the model are compared using the number of parameters and frames per second (FPS) metrics.

### 4.4 Experimental comparison with other models

#### 4.4.1. Experiments with the VisDrone2021 dataset

This research has done experimental validation of several existing better small target detection models on the VisDrone2021-DET-val dataset [6], as shown in Table 1. It can be seen that the ASG-YOLOv5 model designed in this paper is higher than the mAP value of the YOLOv5 model by 2.9%. The accuracy for small target detection is higher than that of the YOLOv5 model by 6.3% of the APS value. The accuracy of the YOLOv5 model is higher by 4.7% and 6.6% for medium and large targets. The ASG-YOLOv5 model has a 1.4% higher AP_val_ value than the YOLOv7 model and a 5.3% higher AP_S_ value than the YOLOv7 model for detection accuracy of small targets. At the same time, this research adds the designed modules into YOLOv7 and YOLOv8 [34]. As shown in Table 1, the detection accuracy of the model designed in this paper is higher than that of ASG-YOLOv7 by 1.3% mAP, although the detection accuracy is slightly higher than that of ASG-YOLOv8, as shown in Table 2, the computation, parameter counts, and speeds of the model designed in this paper are better than those of ASG-YOLOv8, thus proving that the model designed in this paper well balances the speed and detection accuracy and has a large performance advantage. As shown in Table 2, the computational and parametric quantities of the model designed in this paper are relatively low. The FPS value can reach 86, which meets the real-time demand of deploying UAVs for small-target detection. In contrast, the accuracy of the model designed in this paper is much higher than that of the YOLOv7-tiny model, which reflects the balance of small-target detection accuracy and speed of the model designed in UAV scenarios, demonstrating the performance advantage.

**Table 1:** Comparison of model effects on VisDrone2021-DET-val.

Model	AP_val_(%)	AP_50_(%)	AP_75_(%)	AP_S_(%)	AP_M_(%)	AP_L_(%)
Libra R-CNN [35]	14.9	25.2	15.2	5.9	25.6	31.4
RefineDet [36]	14.9	28.8	14.0	-	-	-
RetinaNet [37]	15.0	26.4	15.3	6.3	25.6	34.4
YOLOv3 [38]	15.0	27.2	14.6	6.3	21.5	36.1
DetNet59 [39]	15.2	29.2	14.3	-	-	-
Fcos [40]	17.9	30.4	18.3	9.2	27.6	35.4
ATSS [41]	20.4	33.8	20.9	11.6	31.7	36.7
Tridentnet [42]	19.8	35.0	19.5	11.4	29.6	36.6
TPH-YOLOv5 [3]	19.4	36.4	18.1	11.0	29.4	41.9
DroneEye2020 [43]	17.4	30.6	18.1	10.1	27.2	35.1
DBNet [6]	19.9	36.3	19.1	12.0	29.1	42.4
YOLOv5 [14]	18.2	32.9	17.4	10.4	27.0	35.3
YOLOv7 [44]	19.7	36.7	18.3	11.4	29.3	42.1
ASG-YOLOv7	19.8	36.6	18.2	11.1	28.9	41.3
ASG-YOLOv8	21.0	37.2	20.9	13.2	30.0	41.4
**ASG-YOLOv5(ours)**	**21.1**	**37.7**	**21.0**	**16.7**	**31.7**	**41.9**

**Table 2:** Comparison of the performance of each target detection model at the same level of operation.

Model	Size	#Param.	FLOPs	FPS
YOLOv3	640×640	59.6M	158.0G	27
YOLOv5-S	640×640	7.2M	16.5G	95
YOLOv5-M	640×640	21.2M	49.0G	74
YOLOv7-tiny	640×640	6.2M	13.8G	286
YOLOv8	640×640	14.1M	44.1G	79
**ASG-YOLOv5(ours)**	640×640	**10.1M**	**32.1G**	**86**

In order to realize the real-time performance of the small target detection model in the UAV scenario, this research designed a lightweight module, but there is also a certain number of parameters, which makes the model as a whole increase a certain amount of computational complexity; at the same time, the accuracy of the small target detection in two UAV scenarios has been significantly improved, and shows the advantage of the detection accuracy in the comparison of existing small target detection models, so this research has a better balance between detection real-time speed and detection accuracy. Although the added modules have a certain number of parameters, the accuracy is well improved, which reflects the advantage of the overall performance of the model designed in this paper.

**Fig 8:**
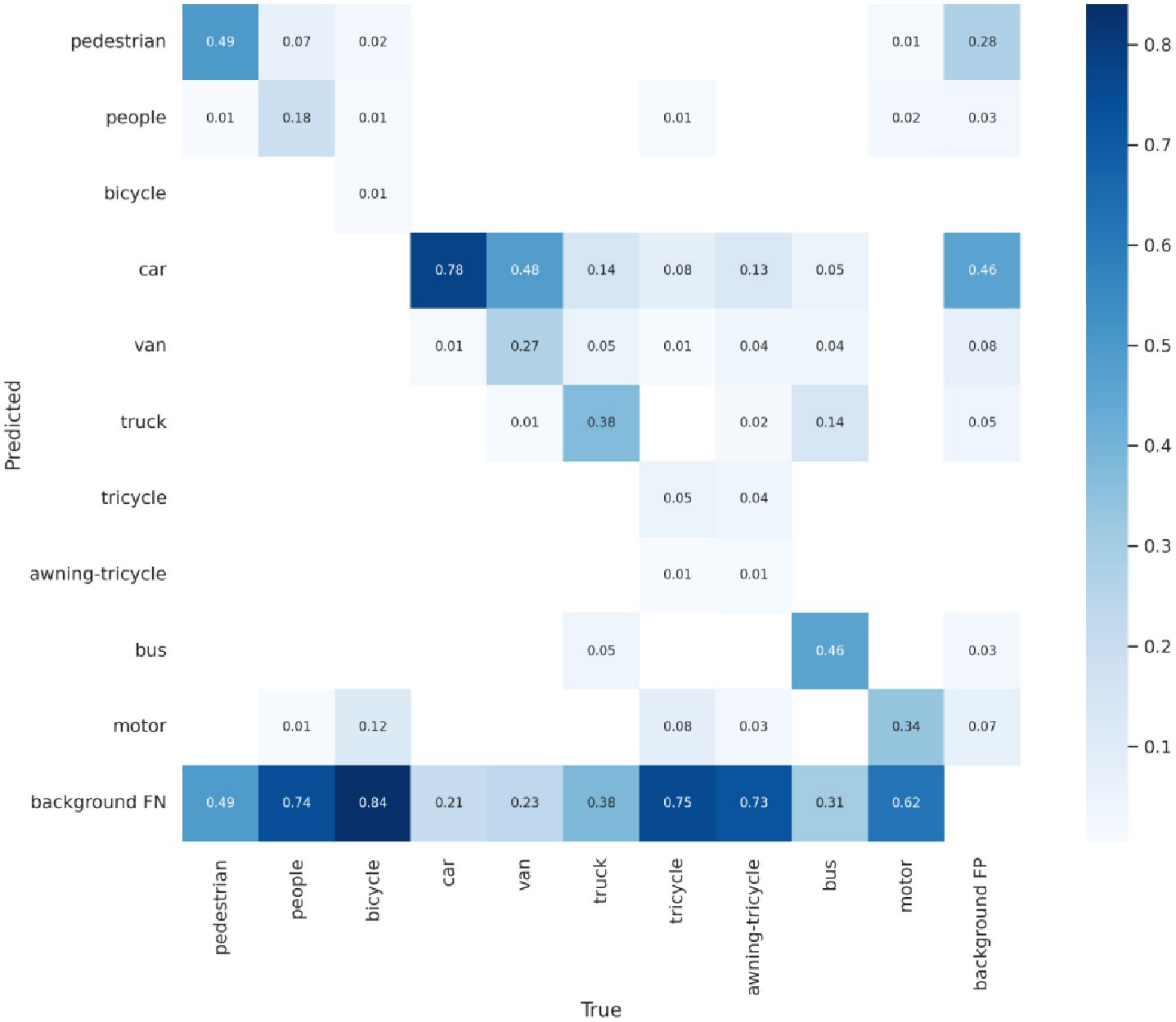
Confusion matrix for the YOLOv5 model on the VisDrone2021 dataset. It contains ten categories of the VisDrone2021 dataset. In the matrix, "background FP" signifies the instances where the model missed detecting non-background category target objects. In contrast, "background FN" indicates the occurrences where the model falsely detected category target objects that were not present.

**Fig 9:**
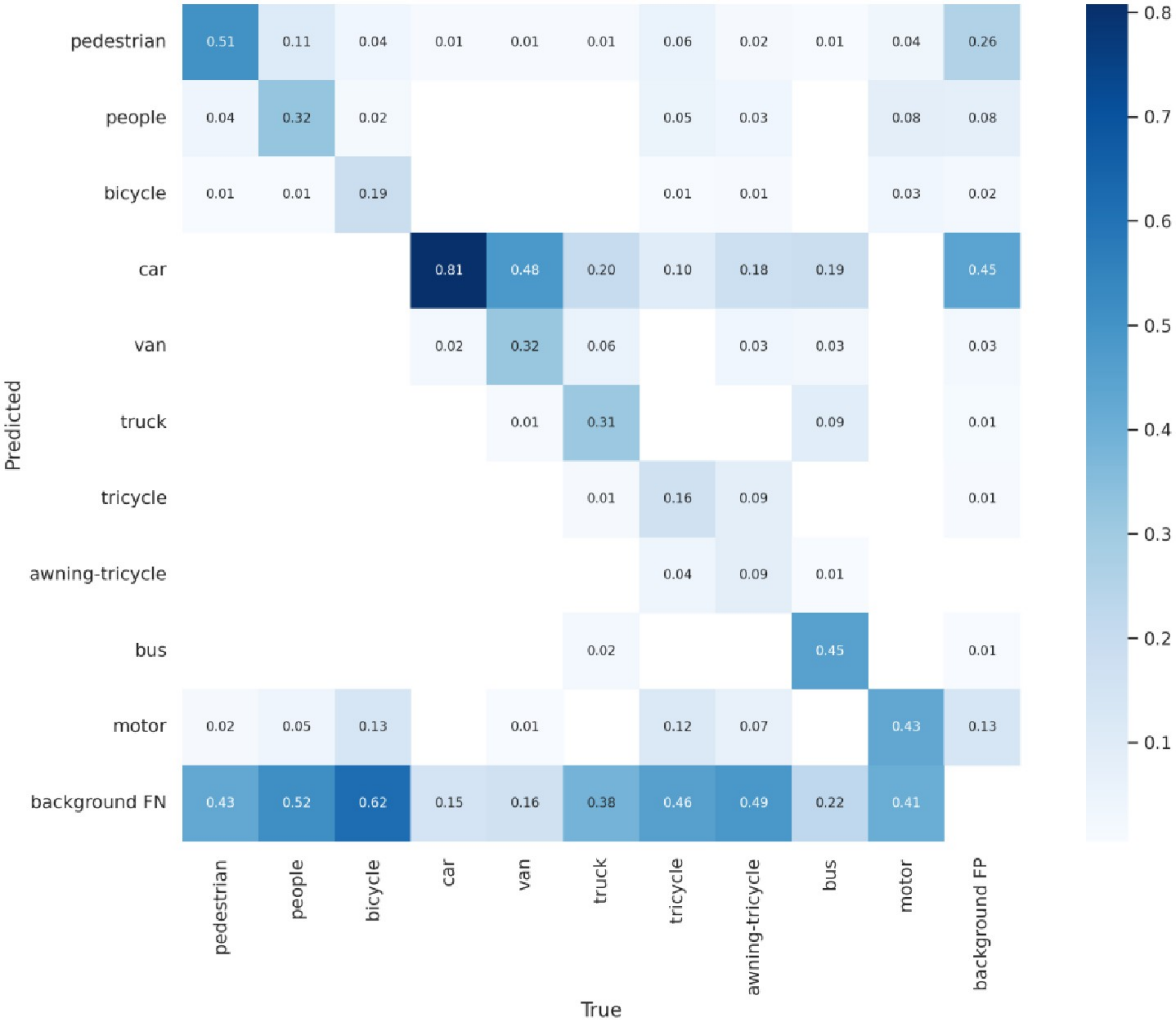
Confusion matrix for the YOLOv7 model on the VisDrone2021 dataset.

**Fig 10:**
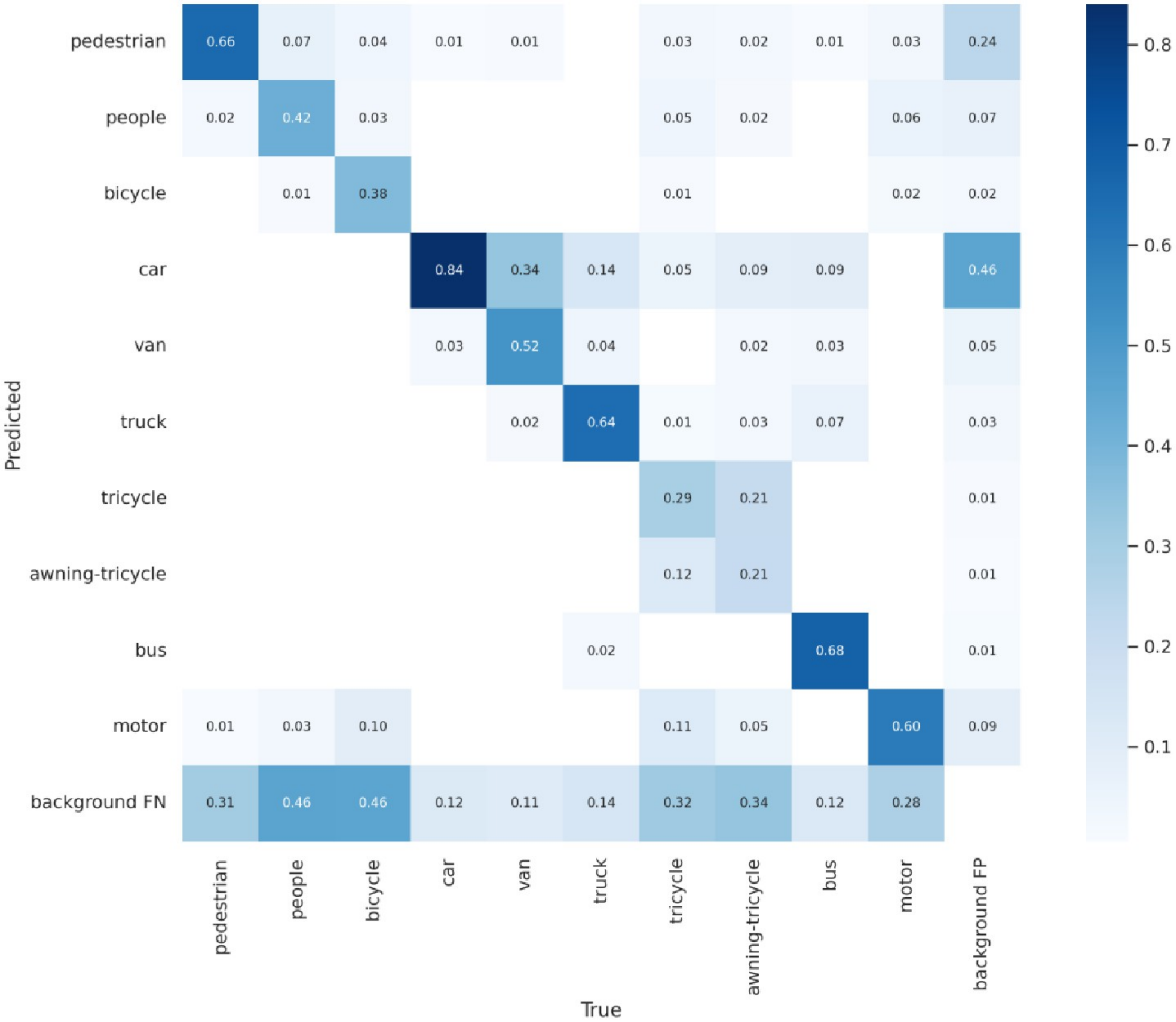
Confusion matrix for the ASG-YOLOv5 model on the VisDrone2021 dataset.

Figs 8–10 display the confusion matrices for the YOLOv5, YOLOv7 models, and ASG-YOLOv5 model, respectively, detecting ten different categories. After being trained, comparing these matrices reveals that the ASG-YOLOv5 model can accurately detect all ten categories in UAV images with higher precision than the YOLOv5 and YOLOv7 models. The ASG-YOLOv5 model scores lower for missed and falsely detected objects than the YOLOv5 and YOLOv7 models, especially for small objects that occur less frequently in certain categories. Notably, the confusion matrix summarizes the object classification performance of the trained models for four different object classes.

Thus, comparing the experimental results, it can be seen that the model designed in this paper has better results on the VisDrone2021 dataset, with a significant improvement in the mAP value, and has a well-balanced performance between the model’s running speed and the number of parameters, which is more adaptable to the detection of small targets under the aerial images of UAVs, and is superior to the majority of the small target detection models.

#### 4.4.2 Experiments with the AI-TOD dataset

This research experimentally validates several existing better small target detection models on the AI-TOD dataset, as shown in Table 3. It can be seen that the ASG-YOLOv5 model designed in this paper has 1.4% higher mAP than the YOLOv5 model. The minimal target detection accuracy is 1.9% higher than the APvt value of the YOLOv5 model, the weak target detection accuracy is 1.7% higher than the APt value of the YOLOv5 model, and the small target detection accuracy is 2.9% higher than the APS value of the YOLOv5 model. For medium-sized targets, the accuracy of the YOLOv5 model is 4.6% higher. Adding the modules designed in this paper to YOLOv7 and YOLOv8, as shown in Table 3, ASG-YOLOv7 and ASG-YOLOv8 are less effective in detecting UAV scenarios compared to the ASG-YOLOv5 model, which is 1.2% mAP higher than the ASG-YOLOv8 model. This indicates that due to the differences in the structure of these three networks, the YOLOv5 network can effectively integrate and utilize the attention and spatial gating mechanisms that this research has designed, but it is not able to adapt or utilize these mechanisms well in the structure of YOLOv7 and YOLOv8; joining to the YOLOv5 network its model can take advantage of its feature expression ability due to the way of feature extraction, the number of layers, and the sensory field, and other factors result in the inability to utilize or adapt this module in YOLOv7 and YOLOv8 fully.

**Table 3:** Comparison of model impacts in the test set of the AI-TOD dataset.

Model	AP_val_(%)	AP_50_(%)	AP_75_(%)	AP_vt_(%)	AP_t_(%)	AP_S_(%)	AP_M_(%)
YOLOv3	4.5	14.2	1.7	2.1	4.6	5.9	6.2
CenterNet [45]	13.4	39.2	5.0	3.8	12.1	17.7	18.9
ATSS	12.8	30.6	8.5	1.9	11.6	19.5	29.2
Tridentnet	7.5	20.9	3.6	1.0	5.8	12.6	14.0
Cascade R-CNN [46]	13.8	30.8	10.5	0.0	10.6	25.5	26.6
DetectoRS w/RFLA [47]	24.8	55.2	18.5	9.3	24.8	30.3	38.2
M-CenterNet [34]	14.5	40.7	6.4	6.1	15.0	19.4	20.4
YOLOv5	24.7	57.9	17.6	10.6	24.4	30.7	35.1
YOLOv8 [34]	25.1	56.2	18.7	8.6	24.3	33.1	39.9
ASG-YOLOv7	24.6	57.4	17.8	10.7	24.7	31.0	35.9
ASG-YOLOv8	24.9	56.1	18.8	8.1	23.6	32.5	39.5
**ASG-YOLOv5(ours)**	**26.1**	**59.8**	**19.6**	**12.5**	**26.1**	**33.6**	**38.7**

#### 4.4.3 Experiments with the NWPU-RESISC dataset

**Fig 11:**
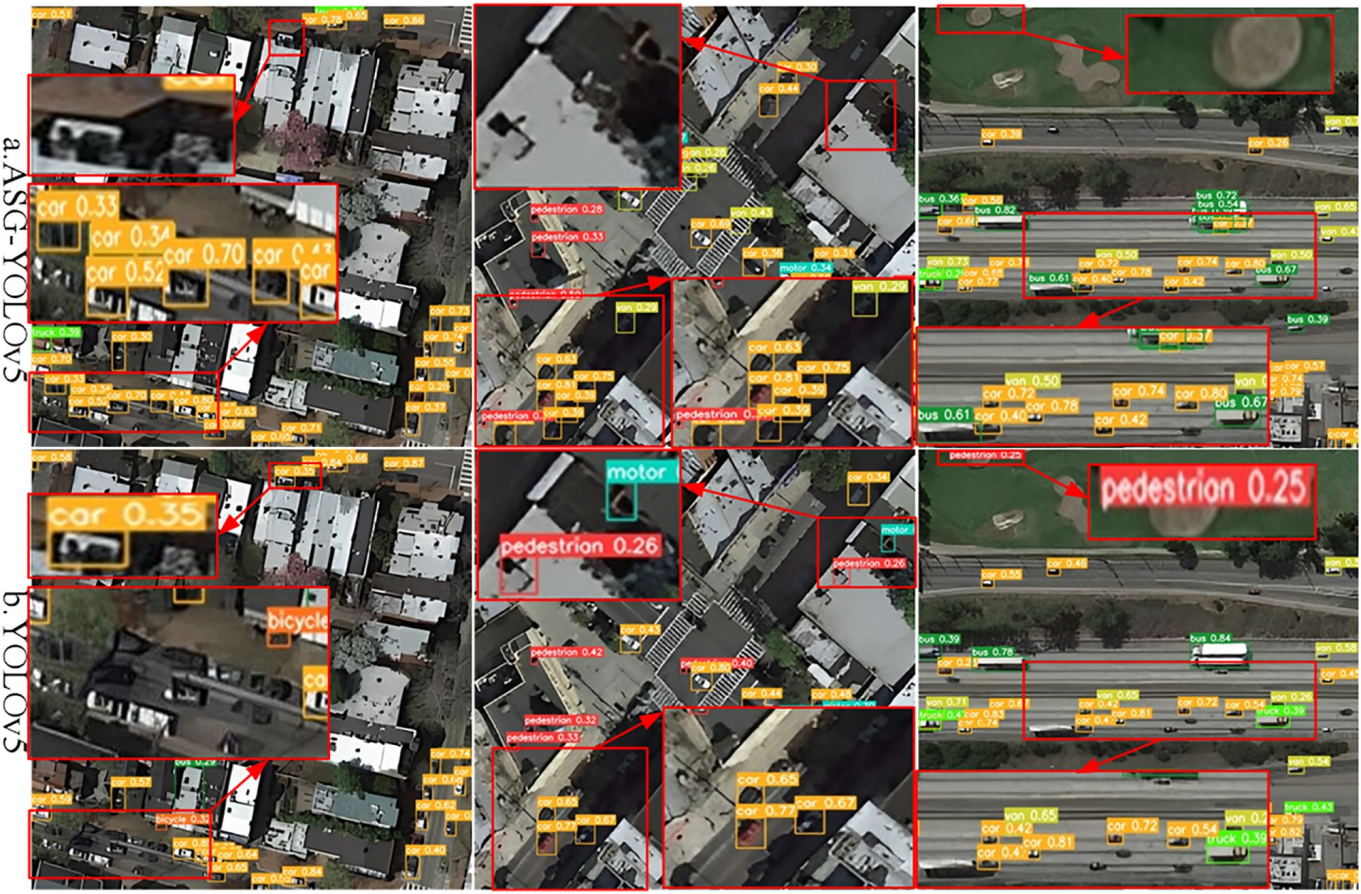
Comparison of ASG-YOLOv5 and YOLOv5 visualizations for detecting various types of weak targets on the NWPU-RESISC dataset, where the targets are identified by the different colors of the marked boxes in different categories in the figure. a. denotes the graph of the visualization effect of the ASG-YOLOv5 model for the UAV remote sensing captured images; b. denotes the graph of the visualization effect of the YOLOv5 model for the UAV remote sensing shooting picture visualization effect diagram. Fig 11 is attributed to the NWPU-RESISC database and are available from the NWPU-RESISC database (url(s) https://tensorflow.google.cn/datasets/catalog/resisc45).

As can be seen from the results of the comparison of the detection detail visualization of the NWPU-RESISC dataset in Fig 11, as well as the detail portions of the red and blue boxes, the ASG-YOLOv5 model in Fig 11a has high confidence in detecting a variety of weak target classes and is able to detect them accurately. In contrast, the YOLOv5 model in Fig 11b suffers from omission problems in detecting target categories. Also, the confidence of target detection is significantly lower than that of the ASG-YOLOv5 model designed in this paper. This verifies that the model designed in this paper is more effective in detecting target objects from tiny to medium target objects in UAV remote sensing captured images with higher detection accuracy.

Overall, it can be seen from the visualization of the NWPU-RESISC dataset detection comparison details in Fig 11 and its details in the red and green boxes that this study is effective in detecting the model in specific scenarios and conditions such as dense targets, light changes, tiny targets, target occlusion, and target motion blurring. Meanwhile, from the comparison of the details in the two figures, it can be seen that the ASG-YOLOv5 model designed in this paper, on the other hand, can be better applied to specific scenes and conditions such as dense targets, light changes (day and night), tiny targets, target occlusion, target motion blurring, etc., and has a better detection ability for capturing tiny targets compared with the YOLOv5 model.

### 4.5 Ablation experiments

On VisDrone2021-DET-val, this research trained different components to analyze different effects, as shown in Table 4.

**Fig 12:**
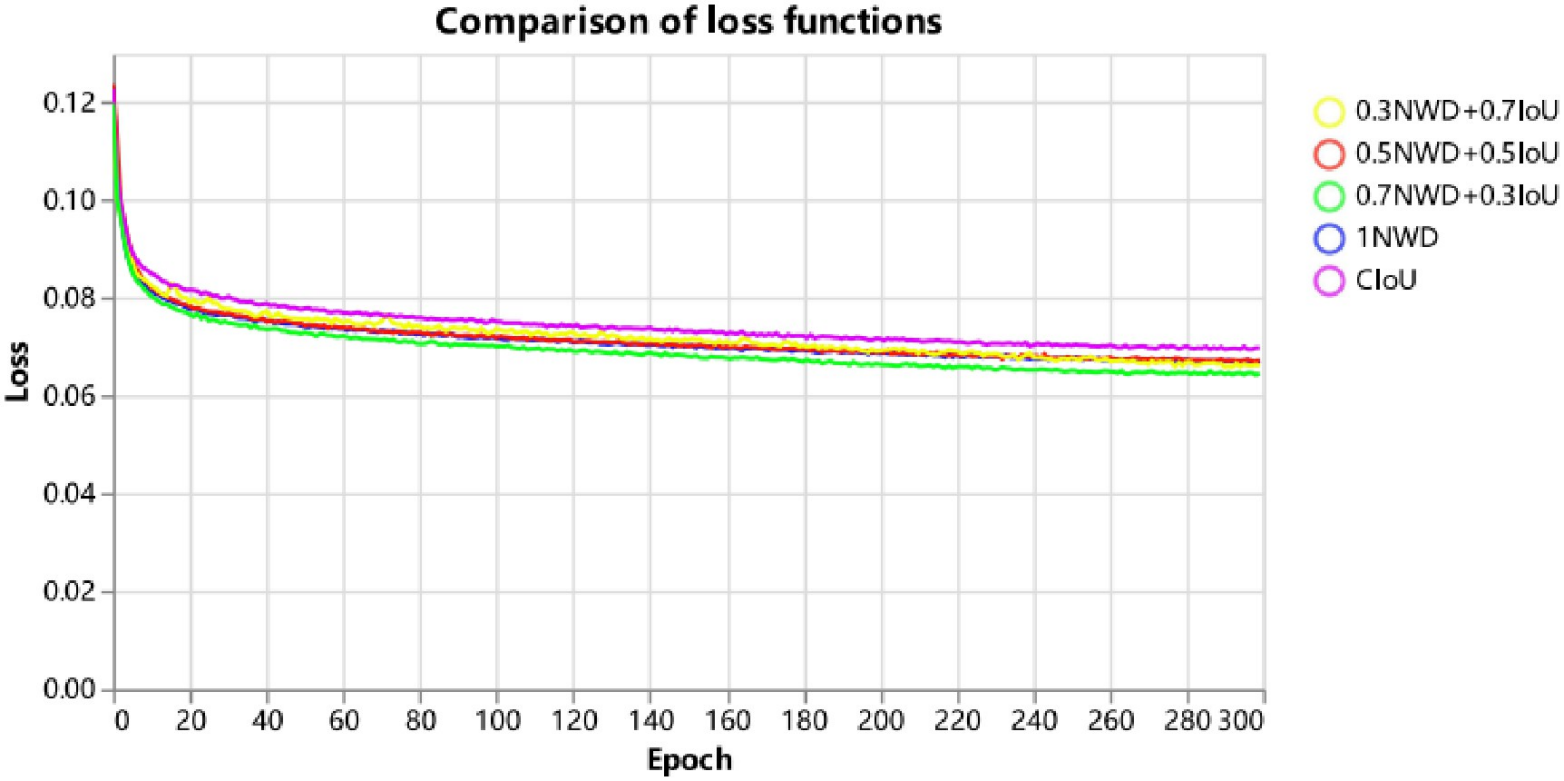
Comparison plots of loss curves for different hyperparameter values of the NWD-CIoU loss function on the VisDrone2021 dataset.

**Fig 13:**
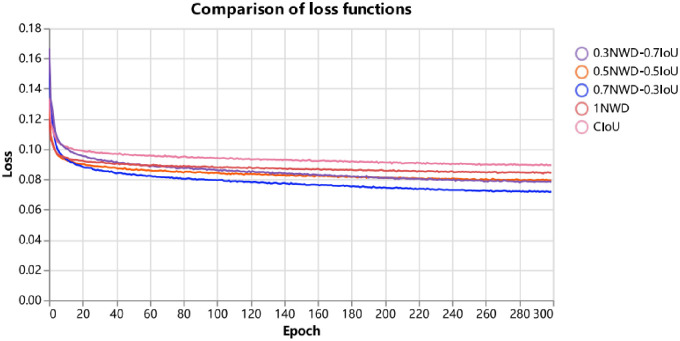
Comparison plots of loss curves for different hyperparameter values of the NWD-CIoU loss function on the AI-TOD dataset.

A. First, the study adds the DCA module to the end of the backbone network of YOLOv5 to compare with the original YOLOv5 model on VisDrone2021-DET-val. As shown in Table 4, it can be seen that the mAP value is improved by 1.5% after adding the DCA module, and from the APS value, it can be seen that the addition of the DCA module improves the model’s focus on small targets, and the detection accuracy is improved by 3.4%. Meanwhile, the study compared the effectiveness and accuracy of Squeeze-and-Excitation Networks and the DCA module designed in this paper for target detection of the YOLOv5 model in UAV filming scenarios, as shown in Table 5, which shows that the mAP value is improved by 1.5% after the addition of the DCA module designed in this paper, which is a good proof of the effectiveness of the DCA module designed in this paper in improving the model’s ability to focus on and detect useful target information. Meanwhile, the DCA module usually needs a large amount of training data to learn effective contextual relationships; however, in domains or tasks such as industry, agriculture, etc., data may be very scarce, limiting the module’s performance. Meanwhile, the DCA module, although lightweight, also needs to compute a large number of attentional weights, which will have a certain number of parameters.
B. Secondly, the study adds the SGM module in the Neck layer of YOLOv5 with the original YOLOv5 model on VisDrone2021-DET-val for comparison. As shown in Table 4, it can be seen that after adding the SGM module, the features extracted by the backbone network are fed into the neck structure, which is filtered by the SGM module on the feature space of the deep feature layer and then combined with the shallow features to introduce the global attentional up-sampling and the weighted fusion of the feature layers of different scales, which enhances the characterization of the useful target categories, reduces the interference of the redundant feature information, and increases the backbone useful feature information in the extracted features of the network. Eventually, the detection accuracy of the model for useful feature targets is improved so that the mAP value of the model is increased by about 1.1%, and it can be seen from the APS value that the model filters out redundant targets and improves the detection accuracy of small targets by 2.6%. The module uses a lightweight structure, and the overall number of parameters of the model has less improvement after the addition of the neck structure. At the same time, the module may be insufficient for eliminating redundant information and feature fusion strategy for small target detection in other scenarios, and the number of parameters of the module still needs to be reduced so that the model can have a faster detection speed in other scenarios.
C. Finally, the study replaced the CIOU regression loss function and added the NWD-CIoU regression loss function. According to Eq 8, the study compared the model detection accuracy of the NWD-CIoU regression loss function on the UAV dataset taking different values of the balance coefficient, as shown in Table 6, the NWD-CIoU loss designed in this paper based on the ASG-YOLOv5 model improves the mAP value of CIOU Loss by 0.3% over CIOU Loss, and the value of the small-target detection accuracy improves by 0.3%; at the same time, balance coefficients taking the values of 0.7NWD and 0.3IoU have the best model detection accuracy. As shown in Figs 12 and 13, the study compared the stability of the model loss function curves under different values of the balance coefficient in the two UAV datasets. When the balance coefficient takes the values of 0.7NWD and 0.3IoU, the loss function curves of the model are the smoothest and the fastest convergence, proving that the loss function’s stability is better in this case.

**Table 4:** Ablation experiments on the VisDrone2021 dataset.

Model	AP_val_(%)	AP_50_(%)	AP_75_(%)	AP_S_(%)	AP_M_(%)	AP_L_(%)
YOLOv5	18.2	32.9	17.4	10.4	27.0	35.3
+DCA	19.7(+1.5)	35.1	19.1	13.8	29.5	38.6
+SGM	20.8(+1.1)	37.5	20.3	16.4	31.6	41.5
+NWD-CIoU Loss	21.1(+0.3)	37.7	21.0	16.7	31.7	41.9

**Table 5:** Attention mechanism ablation experiments on the VisDrone2021 dataset.

Model	AP_val_(%)	AP_50_(%)	AP_75_(%)	AP_S_(%)	AP_M_(%)	AP_L_(%)
YOLOv5	18.2	32.9	17.4	10.4	27.0	35.3
+SENet	18.4(+0.2)	32.9	18.2	10.9	26.6	35.0
+DCA	19.7(+1.5)	35.1	19.1	13.8	29.5	38.6

**Table 6:** Loss function ablation experiments on the VisDrone2021 dataset.

ASG-YOLOv5	AP_val_(%)	AP_50_(%)	AP_75_(%)	AP_S_(%)	AP_M_(%)	AP_L_(%)
+CIoU	20.8	37.5	20.3	16.4	31.6	41.5
+0.3NWD+0.7IoU	20.2	36.8	18.9	10.9	28.3	38.1
+0.5NWD+0.5IoU	20.9	37.3	20.5	16.5	31.4	41.3
+1NWD	20.6	37.1	20.0	15.8	30.9	40.5
+0.7NWD+0.3IoU	21.1(+0.3)	37.7	21.0	16.7	31.7	41.9

## 5 Conclusion

In this paper, for the problem of small target detection in unmanned aerial vehicle remote sensing aerial images, the study proposes the ASG-YOLOv5 model by taking YOLOv5 as the base model, adding some cutting-edge target detection techniques and improving the backbone network and neck structure of YOLOv5. The study designs a dynamic contextual attention module, which is added to the end of the backbone network to dynamically assign target weights and fuse global and local contextual information to improve the model’s attention to small target feature information in the extracted features. In the multi-scale fusion stage, this research designed spatial gated filtering multi-directional weighted fusion module to reduce the interference of redundant features in the features extracted from the backbone network and to increase the information of small target pixels in the underlying feature layer so that the model can better extract useful target features; the introduction of the Normalized Wasserstein Distance improves the regression loss function, measures the similarity of Gaussian distributions between regression frames, and increases the ability to measure the similarity between mutually inclusive bounding boxes and the smoothing of small target differences. On the VisDrone2021 and AI-TOD datasets, the model designed in this paper has been subjected to several comparative experiments with other newer small target detection models, achieving better detection accuracy and significant results in applying attention and spatial gating mechanisms. The combination with different state-of-the-art techniques makes the improved YOLOv5 model more suitable for detecting small targets in UAV remote sensing aerial photography images. It also solves the problems of tediousness, scale uncertainty, and environmental changes when there are multiple object classes in different scale images in UAV remote sensing aerial photography and better prevents the phenomenon of missed detection. By optimizing and adjusting for UAV scenarios, an effective solution is provided for this UAV small target detection field, which promotes the development of small target detection technology and improves the effectiveness of UAV missions. However, the method in this paper still has some limitations; although the lightweight module is added, the overall parameter count of the model still needs to be increased, which makes the model slow down in the application scenarios. Meanwhile, the model designed in this paper and existing models still have low detection accuracy for small targets in UAV scenarios. This study will further investigate the limitations of the improved model and apply the model to scenarios such as image segmentation.

## Supporting information

S1 File Dataset. (DOCX)
