# Supplementary material for: ASG-YOLOv5: Improved YOLOv5 unmanned aerial vehicle remote sensing aerial images scenario for small object detection based on attention and spatial gating
Source: PLoS One. 2024 Jun 3;19(6):e0298698. doi: 10.1371/journal.pone.0298698 (PMC11146694; doi:10.1371/journal.pone.0298698)
Supplement: S1 File — (DOCX) [file pone.0298698.s001.docx]

**Supporting information**

In our experiments, we extracted images of unmanned aerial vehicle remote sensing aerial scenes from NWPU-RESISC datasets to perform related experiments.

The datasets can be downloaded from the following link:

NWPU-RESISC dataset https://tensorflow.google.cn/datasets/catalog/resisc45.
